# Predicted *Batrachochytrium dendrobatidis* infection sites in Guyana, Suriname, and French Guiana using the species distribution model maxent

**DOI:** 10.1371/journal.pone.0270134

**Published:** 2022-07-14

**Authors:** Jairam Rawien, Sabitrie Jairam-Doerga

**Affiliations:** 1 Anton de Kom University of Suriname, National Zoological Collection Suriname, Paramaribo, Suriname; 2 Anton de Kom University of Suriname, National Herbarium of Suriname, Paramaribo, Suriname; University of Molise, Isernia, ITALY

## Abstract

The fungal pathogen *Batrachochytrium dendrobatidis* (Bd) which causes that amphibian disease chytridiomycosis is expanding its worldwide range from an Asian origin, infecting amphibians in a growing number of countries. Modelling the potential range of this amphibian pathogen using environmental variables and presence data could advance our understanding of at-risk areas and species in locations with limited surveillance to date. We used a species distribution model to assess Bd habitat suitability in the three Guiana’s (Guyana, Suriname, and French Guiana) in South America. The model output showed that all three countries have substantial areas where Bd could grow and proliferate, and maximum temperature of the warmest month was the top predictor of suitable Bd habitat, inversely correlated with modeled Bd occurrence. Predicted Bd infection areas in Guyana and French Guiana were large and localized whereas possible sites in Suriname were more scattered throughout the country. The areas projected as potential suitable in Suriname were mostly high elevation regions. These results could help inform efficiencies for development of a proactive monitoring program that could alert managers of novel Bd outbreaks for focused mitigation actions to forestall the spread of this amphibian disease.

## Introduction

It is no secret that biological diversity worldwide is in peril and is being eradicated to such an extent that we can now speak of a global sixth extinction event [1–4]. Estimated species numbers have decreased approximately 4000 to 6000 species per year, if not more, due to rainforest deforestation alone [5, 6]. Amphibians are among the top taxa affected by global losses, with 40% of species threatened with extinction (IUCN 2020: https://www.iucn-amphibians.org/; accessed 14 September 2020) from threats including extensive habitat loss [7, 8] and disease such as the amphibian chytrid fungus *Batrachochytrium dendrobatidis* (Bd) [9, 10]. Although Bd can be found in many countries worldwide [11, 12], it has a climate niche where optimal growth has been observed at moderate temperatures (e.g., to 28°C: [13]; 7 to 25°C: [14]. Areas with moderate temperatures in the tropics have included mountainous regions with a relatively high altitude [15], although amphibian species occurring in non-mountainous areas less than 100m ASL have developed the disease [16–18]. Additionally, global metadata analyses of Bd occurrences defined the Bd climate-niche space with top predictors including temperature range at a site, and the low, mean, and maximum monthly average maximum temperature at sites [19]. Occurrences are also tied to human-mediated transmission [20–24], and as an aquatic invasive species, it is unlikely to have reached stable equilibrium in suitable habitat. In South America, Bd has been found in 10 of 13 countries (Bd-maps database, accessed 08 August 2020). The three Bd-free countries are Paraguay, Guyana and Suriname [25], the last two of which are part of a unique Precambrian geological formation–the Guiana Shield–which also includes French Guiana. For the three Guiana’s (Guyana, Suriname and French Guiana), Bd occurs in French Guiana, where it was first detected in 2012 [26] and more widespread after surveying other regions in French Guiana [27]. Having an almost similar climate and geography coupled with the frequent human travel between these three countries it might only be a matter of time for amphibians in Guyana and Suriname to become Bd infected. Species Distribution Models (SDMs) are an ideal tool to assess suitable habitat areas that might be especially vulnerable to Bd occurrences (e.g., continental analyses: [11, 19, 28, 29]. To date, no study has solely applied SDMS to the downscaled landscape of Guyana, Suriname and French Guiana. We used the SDM maxent to develop a Bd habitat suitability model for the three Guianas, using world climate data at a relatively fine-resolution spatial grid of 30 seconds (1 x 1km^2^) latitude and longitude. Our aim was to assess where in the Guianas anurans could be at risk of Bd infection, to inform the proactive development of a monitoring program to detect possible Bd incursion. Although models depicting areas that are going to be affected are informative, living in Suriname we also wanted to know what areas in Suriname would be specifically affected. Suriname has been, apart from French Guiana the only country of the 3 Guiana’s to have done multiple tests for Bd. This was done both on museum specimens and specimens in the field [25, 30]. This study will be a completion of both aforementioned studies and allow all those involved to take precautionary measures in keeping Bd at bay.

## Materials and methods

**Fig 1:**
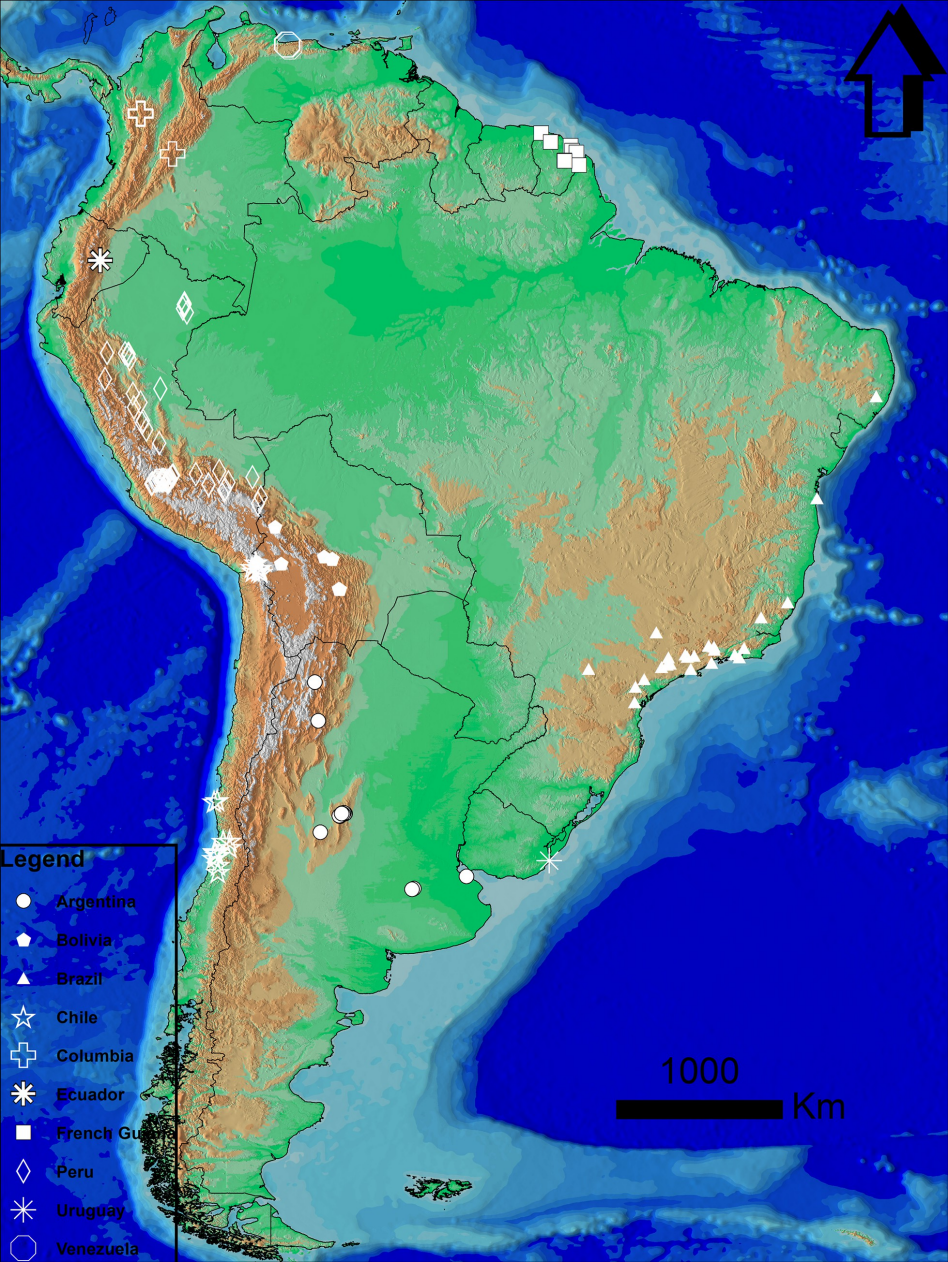
Overview of the presence only (P.O) points used in this study. These points were used to model and predict *Batrachochytrium dendrobatidis* habitat in South America and more specifically in Guyana, Suriname and French Guiana. Coordinates of P.O points are provided in S1 Fig.

Due to the scarcity of Bd-positive data from the Guianas alone we started by modelling the potential distribution of Bd in South America using Bd-positive detections sampled from across the continent until January 2020 (Bd maps.net). For quality control, we removed data records with imprecise and unverified coordinates, retaining data with GPS coordinates taken during Bd sampling. We used presence-only (P.O.) data to refrain from incorporating false negatives which can be a substantial part of Bd histological assays [31] and affect detectability of the pathogen across different temperature gradients [32, 33]. After these screens, the resulting 191 data records were Bd-positive samples from host anuran species distributed in various locations in Argentina, Bolivia, Brazil, Chile, Colombia, Ecuador, Peru, Uruguay, Venezuela and French Guiana (Fig 1). These records were then added to the species distribution modeling software Maxent [34]. Maxent is a machine learning algorithm that uses presence data and random background points to predict the distribution of maximum entropy correlated to certain environmental variables. The outcome shows the habitat suitability for a particular species [34–36]. When the data for modelling is scarce (as in our case for the 3 Guiana’s) Maxent has demonstrated its capability in building reliable models and aptly tweaked to utilize the available data at its maximum [37–39]. Bioclimatic variables with a spatial resolution of 30 s (~ 1 km^2^) were batch downloaded from Worldclim.org v2.1 (1970 to 2000) [40] and extracted for our region using ArcGIS v10.4. Informed by previous studies [13, 19, 28, 41–44], we used six bioclimatic variables in our model: annual mean temperature; maximum temperature of the warmest month of the year; minimum temperature of the coldest month; annual precipitation; precipitation of the wettest month; and precipitation of the driest month. To overcome the sampling bias of our data [45] we used the method by [46] Boria et al. to spatially filtered data to create a bias file. Spatially filtering data has shown to significantly improve the predictive performance of maxent [46, 47]. Bias files are necessary due to researchers sampling in areas that are easily accessible creating geographic locality clusters [48, 49]. To create a bias file from our dataset we used ArcGIS v10.4 to create a buffer of 5 km around each Bd-positive record. From overlapping 5 km clusters one point was arbitrarily selected to be used as training data in maxent. The remaining points within that overlapping buffer were then used as the bias file in maxent. This resulted in 100 unique points which were used for the training dataset and the bias file S1 Table. We ran the maxent software using auto features selected. We selected the variable jackknife calculation in the maxent software which shows the contribution of each bioclimatic variable to the model. To achieve the most accurate results the model was run using 10.000 background points [50] and a maximum of 500 iterations. The data was then run a 100 times (replicates) randomly using different test samples (random seed) set at 25 percent of the P.O. data for each run. We chose the default cloglog output function because of its ability to visually have a stronger difference for areas with moderately high output [35]. The results from the 100 replicates are shown in minimum, average and maximum maps showing the habitat suitability for Bd for south America and specifically for the three Guianas. This allowed us to distinguish the predicted habitat suitability per country. Focusing specifically on Suriname on the areas that were predicted to be affected by Bd we georeferenced the maxent output file and added an ArcMap shape file with the elevation for the areas affected in Suriname. This would allow us to specifically identify the areas that are highlighted as potential Bd sites.

## Results

**Fig 2:**
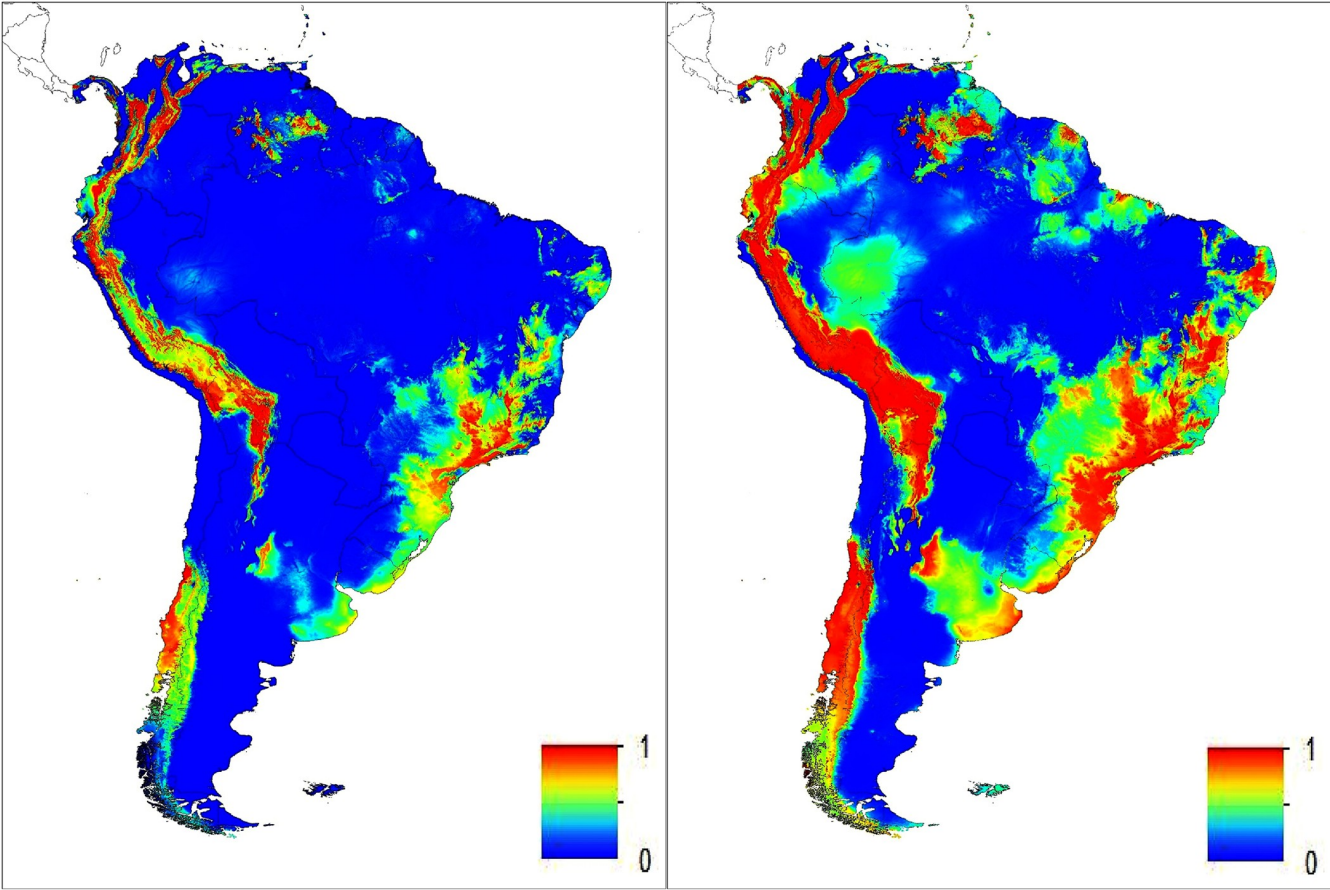
Overview of areas to be affected by *Batrachochytrium dendrobatidis* (Bd) predicted by the species distribution model maxent using Worldclim variables and Bd presence data throughout South America. Figure on the left shows the minimum suitability for Bd whilst the figure on the right shows the maximum projected predictions for Bd. Warmer colors indicate a higher suitability of the area for Bd infection.

**Fig 3:**
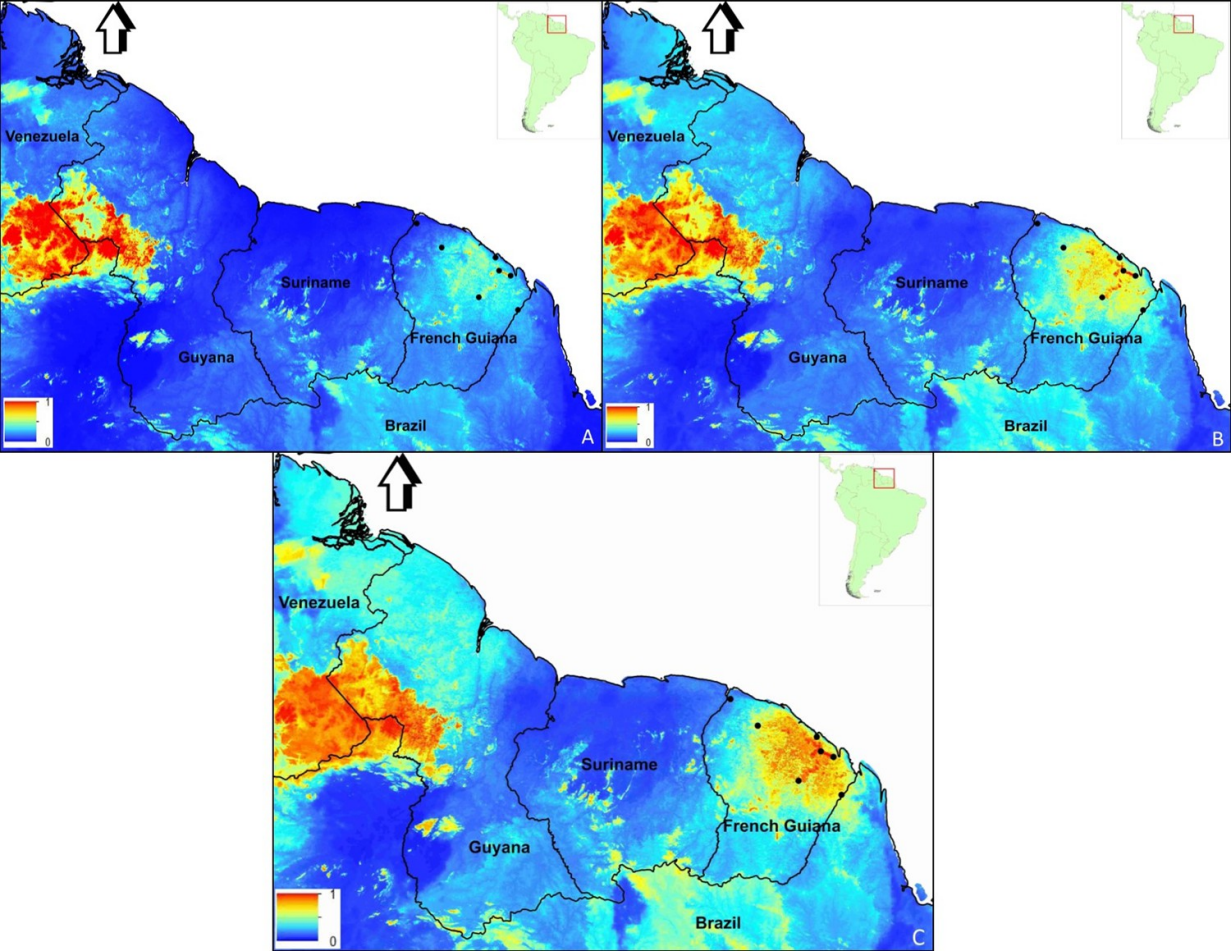
Potential distribution of the chytrid fungus *Batrachochytrium dendrobatidis* (Bd) in the three Guianas, projected using maxent models of six climate metrics. A) the minimum distribution; B) median distribution; C) the maximum distribution. Warmer colors indicate a higher suitability of the area for Bd infection. Black points are Bd positive sites in French Guiana.

**Fig 4:**
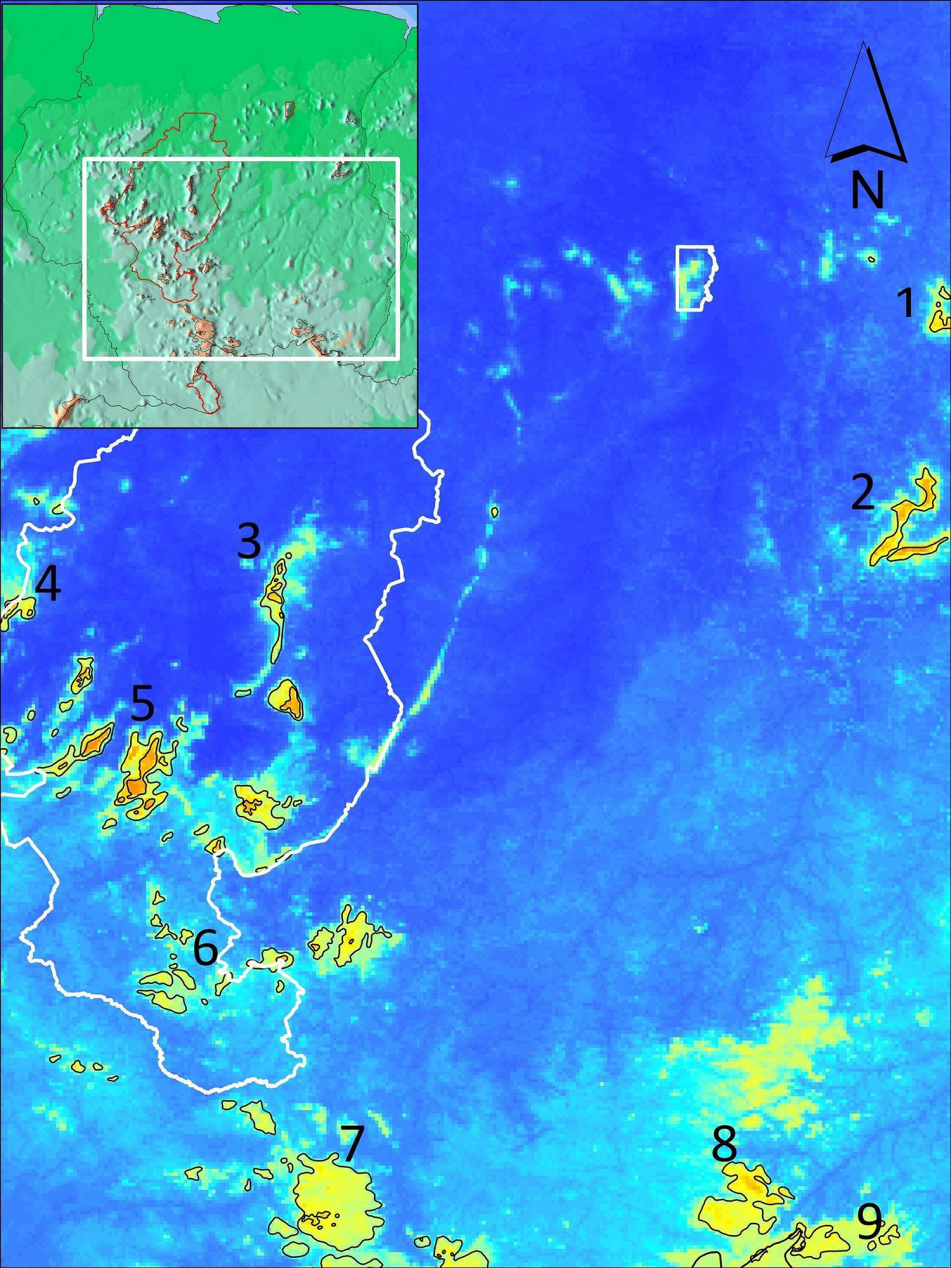
Overview of the areas in Suriname that are projected as viable for the fungus *Batrachochytrium dendrobatidis*. Warmer colors indicate higher chances of possible infection. Numericals are for areas listed as follow: (1) Nassau mountain, (2) Lely mountain, (3) Emma mountain range, (4) Bakhuis mountain, (5) Wilhelmina mountain range, (6) Eilerts de Haan mountain range, (7) Grens mountain range, (8) Oranje mountain and lastly (9) Tumuk Humak mountain range.

The output of the maxent model supported ‘good values’ of our data as defined by Swets [51]. The omission on test samples was a very good match to the predicted omission rate. To further assess the performance of the SDM model, the area under the receiver operating characteristic curve (AUC) was 0.831, an indication that the model performed well in predicting suitable habitats for Bd [51]. AUC values closer to 0.5 indicate that the performance of the model is not better then random whereas a value closer to 1 indicates better model performance [52], adapted from Swets [51]. We found that the maximum temperature of the warmest month of the year was the most significant factor in predicting Bd suitable habitat (64.9%). The second variable that came relatively near in having some impact on Bd predictability was precipitation of the wettest month, although at a much lesser percentage of 20.5%. All other environmental factors (annual mean temperature; minimum temperature of the coldest month; annual precipitation and precipitation of the driest month) were below 10% and contributed very little to the model. To further estimate the importance of variables we also had maxent calculate a jackknife test. From the jackknife test we also found that the environmental variable with the highest gain when used in isolation was the maximum temperature in the warmest month, which therefore appears to have the most useful information by itself. That is also the environmental variable that decreases the gain the most when omitted, which therefore appears to have the most information that isn’t present in the other variables. The same pattern is evident when using the jackknife on the test gain instead of training gain; and when using AUC on test data, the maximum temperature in the warmest month had the most information when used in isolation. An overview of the acquired output for South America in terms of Bd habitat suitability showed several likely Bd-hotspot areas (Fig 2). When zooming into the three Guianas, maxent’s SDM predicted different clusters of Bd suitable habitat by country (Fig 3A–3C). For Guyana, a large area of suitable Bd habitat was found in the northwestern part of the country. This Bd-suitable area in Guyana is present in the minimum projection by maxent and continues to ‘intensify’ as a high-risk area for Bd infection in the median and maximum projected maps. After georeferencing we found this area to be mainly composed of elevations ranging from 500 m to 1500 m. Several other smaller fragmented areas of high Bd risk occur throughout Guyana. Aforementioned areas are congruent with areas in Venezuela that are projected as Bd possible locations which also consists of high elevation sites. In Suriname, we found Bd infection likelihood in the southern region (Fig 4). All areas are mountainous regions with a high suitability found in the Central Suriname Nature Reserve (CSNR). This reserve is the largest nature reserve in Suriname. Some of the areas highlighted are the Emma mountain range, the Wilhelmina mountain range, the Asch van Wijck mountain range, the Eilerts de Haan mountain range and lastly the Bakhuis mountains. In the southern region of Suriname, we find mountain ranges predicted as suitable which are on the border with Brazil. These areas show border overlapping suitability with Brazil. French Guiana was the only country of the three Guianas where Bd detections have been found in several places [26, 27]. From Fig 3 we can deduce that the northeastern part of the country is most likely to be affected by Bd. This also coincides with the sites where Bd-positive samples were detected by Courtois et al. [26, 27]. Different from Guyana and Suriname is that the projected area suitable as Bd habitat is mostly below 300 m altitude, whereas Guyana and Suriname have projected Bd-occupied areas mostly higher than 500 m. Fewer scattered areas in the central part of French Guiana with an elevation above 500 m are also predicted to be potential areas for Bd. After plotting all positive locations used to do the modelling of Bd habitat we found this study to accurately predict 96.8% of Bd affected areas based on the data (S1 Table and S1 Fig).

## Discussion

The study done herein to predict Bd habitat for the 3 Guiana’s is very modest in terms data used when compared to other studies predicting Bd suitability areas [19, 28]. However, when examining the effect of the data and the modelling put together we find the model accurately predicting Bd positive sites in South America. We used Bd predictor variables (annual mean temperature; maximum temperature of the warmest month of the year; minimum temperature of the coldest month; annual precipitation; precipitation of the wettest month; and precipitation of the driest month) that mattered the most in being able to show the possible sites in danger of being infected. The results from this show that maximum temperature of the warmest month of the year is most effect in predicting Bd and this has also been shown by other studies such as Rödder et al. [42], Whitfield et al. [53]. Our modelling of the habitat suitability for Bd in the Guianas has shown these countries to have significant large areas that are potentially suitable habitat for this disease-causing pathogen. In Guyana and French Guiana, we observed that most of the projected ‘high risk’ areas were clustered in certain parts of the country whereas for Suriname, suitable sites were found to be more scattered throughout the southern part of the country. From the projected Bd suitability maps for the three Guianas we found a significant chance of eventually acquiring Bd infections within their borders, based on the environmental variables we assessed. Guyana has no recorded Bd as yet, but borders adjacent Venezuela which has already noted several Bd-positive cases [54, 55]. The predicted area for Guyana has shown that Bd is most likely to occur at higher altitude regions. Areas of higher altitude with lower temperatures have been mentioned in several studies as one of the key factors contributing to Bd infections [15, 16, 41]. This however is not the case for French Guiana. Here the projected maps show Bd suitable habitat closer to 300 m and below 30 m ASL. Bd-positive frogs have not been detected in Suriname to date [25, 30, 56, 57]. At a coarse spatial scale, our results somewhat coincide with Ron [28] and Xie et al. [19] although these studies used a different software program at a coarser spatial resolution. The Bd-habitat maps produced herein provide a more in-depth overview of the areas where host anurans are probably at risk of being Bd-infected. This is due to the finer resolution of the environmental layers used in modeling and by the inclusion of the Bd-positive locations in French Guiana data that was previously unused in similar modelling studies.

## Conclusion

Predicting the possible sites of Bd infection for Guyana, Suriname and French Guiana will allow us to fine-tune the geographic scope of surveillance efforts for more efficient monitoring. Our spatial prediction also has allowed us to narrow the likely amphibian taxa in danger of getting infected, given known species occurrences overlapping modeled habitat areas. The situation in French Guiana concerning Bd has shown us that no country is actually totally safe to this fungus. Till date no immediate cause has been mentioned for French Guiana as the source of infection which first occurred in the dendrobatid genus *Dendrobates tinctorius* [26]. With aforementioned species occurring in both Suriname and Guyana, as well as other members of the dendrobatid group, we should urge those concerned to take proactive measures well in advance. This is especially true as no specific causative reason is mentioned for the sudden Bd outbreak in French Guiana. For Suriname field surveys have been done in different parts of the country. A historical assessment of possible Bd presence in museum specimens from Suriname further complemented aforementioned field surveys, although no positive Bd specimens were detected. Similar surveys are recommended for Guyana as well to assess the possibility of Bd presence beforehand.

## Supporting information

S1 Table Overview of the presence only points used for this study. Data used to predict *Batrachochytrium dendrobatidis* habitat in South America. Doi: 10.6084/m9.figshare.20024468.
(DOCX)

S1 Fig Model validation. Overview of the presence only points used for this study as plotted by maxent Doi: 10.6084/m9.figshare.20024540.
(TIF)
