# Supplementary material for: Predicted Batrachochytrium dendrobatidis infection sites in Guyana, Suriname, and French Guiana using the species distribution model maxent
Source: PLoS One. 2022 Jul 14;17(7):e0270134. doi: 10.1371/journal.pone.0270134 (PMC9282542; doi:10.1371/journal.pone.0270134)
Supplement: S1 Table — Data used to predict Batrachochytrium dendrobatidis habitat in South America. Doi: 10.6084/m9.figshare.20024468. (DOCX) [file pone.0270134.s001.docx]

| **Country** | **Location** | **Latitude** | **Longitude** |
| --- | --- | --- | --- |
| Argentina | Punta Lara Natural Reserve, Ensenada | -34.8033 | -58.0099 |
| Argentina | 9 de Julio City, Buenos Aires Province, semi natural permanent pond near town | -35.4869 | -60.90011 |
| Argentina | 9 de Julio City, Buenos Aires Province, ditch in town | -35.4534 | -60.86589 |
| Argentina | 9 de Julio City, Buenos Aires Province, natural permanent pool in nearby rural area | -35.5028 | -60.95489 |
| Argentina | Toro Muerto stream, locality Villa Flor Serrana, Cordoba province | -31.3967 | -64.5936 |
| Argentina | Toro Muerto stream, locality Villa Flor Serrana, Cordoba province | -31.3855 | -64.6057 |
| Argentina | Los Patos, Salta Province | -24.2472 | -66.23275 |
| Argentina | Strict Nature Reserve "Quebarada de las Higueritas", Luján, San Luis Province | -32.403 | -65.927 |
| Argentina | Strict Nature Reserve "Quebarada de las Higueritas", Luján, San Luis Province | -32.403 | -65.927 |
| Argentina | Strict Nature Reserve "Quebarada de las Higueritas", Luján, San Luis Province | -32.403 | -65.927 |
| Argentina | Strict Nature Reserve "Quebarada de las Higueritas", Luján, San Luis Province | -32.403 | -65.927 |
| Argentina | tributary of Rio Los Zarzos, El Pichao, Tucumán Province | -26.3533 | -66.04589 |
| Argentina | Los Gigantes 2 | -31.3932 | -64.7757 |
| Argentina | Cerro Blanco | -31.3543 | -64.653992 |
| Argentina | Copina | -31.4042 | -64.709372 |
| Argentina | Rio Yuspe | -31.3802 | -64.772783 |
| Argentina | Rio Yuspe | -31.3802 | -64.772783 |
| Argentina | La Ventana | -31.5277 | -64.868233 |
| Argentina | Los Gigantes 1 | -31.4011 | -64.787333 |
| Argentina | Los Gigantes 1 | -31.4011 | -64.787333 |
| Argentina | El Volcan | -31.5067 | -64.898508 |
| Argentina | La Ventana | -31.5277 | -64.868233 |
| Argentina | El Volcan | -31.5067 | -64.898508 |
| Argentina | El Volcan | -31.5067 | -64.898508 |
| Argentina | Los Gigantes 2 | -31.3932 | -64.7757 |
| Bolivia | stream between Totora and Huayllamarca, Prov. Carangas, Dept. Oturo | -17.8129 | -68.0501 |
| Bolivia | stream between Totora and Huayllamarca, Prov. Carangas, Dept. Oturo | -17.8129 | -68.0501 |
| Bolivia | between Curahuara of Carangas and Totora, Prov. Carangas, Dept. Oturo | -15.8209 | -68.3751 |
| Bolivia | Sehuencas, Prov. Carrasco, Dept. Cochabamba | -17.5444 | -65.2683 |
| Bolivia | 500 m E of Jatun Pino, Prov. Carrasco, Dept. Cochabamba | -17.5682 | -65.2939 |
| Bolivia | Sehuencas, Prov. Carrasco, Dept. Cochabamba | -17.5444 | -65.2683 |
| Bolivia | Sehuencas, Prov. Carrasco, Dept. Cochabamba | -17.5444 | -65.2683 |
| Bolivia | Rio Casapilla, detour to Khauniri, Prov. Pacajes, Dept. La Paz | -17.7096 | -69.3496 |
| Bolivia | Tiraque, Prov. Tiraque, Dept. Cochabamba | -17.4277 | -65.7185 |
| Bolivia | Tarabuco, Prov. Yamparaez, Dept. Chuquisaca | -19.1762 | -64.9175 |
| Bolivia | between Tarabuco and Icla, Prov. Yamparaez, Dept. Chuquisaca | -19.2045 | -64.855 |
| Bolivia | between Tarabuco and Icla, Prov. Yamparaez, Dept. Chuquisaca | -19.2045 | -64.855 |
| Brazil | Itacaré, BA | -14.2767 | -38.998846 |
| Brazil | Itacaré, BA | -14.2767 | -38.998846 |
| Brazil | Itacaré, BA | -14.2767 | -38.998846 |
| Brazil | RPPN - Capitão, Itacaré, BA | -14.2767 | -38.998846 |
| Brazil | RPPN - Capitão, Itacaré, BA | -14.2767 | -38.998846 |
| Brazil | Sítio dos Boza, Santa Teresa, ES | -19.9315 | -40.595243 |
| Brazil | Sítio dos Boza, Santa Teresa, ES | -19.9315 | -40.595243 |
| Brazil | Sítio dos Boza, Santa Teresa, ES | -19.9315 | -40.595243 |
| Brazil | Sítio dos Boza, Santa Teresa, ES | -19.9315 | -40.595243 |
| Brazil | Sítio dos Boza, Santa Teresa, ES | -19.9315 | -40.595243 |
| Brazil | Sítio dos Boza, Santa Teresa, ES | -19.9315 | -40.595243 |
| Brazil | Sítio dos Boza, Santa Teresa, ES | -19.9315 | -40.595243 |
| Brazil | Sítio dos Boza, Santa Teresa, ES | -19.9315 | -40.595243 |
| Brazil | Sítio dos Boza, Santa Teresa, ES | -19.9315 | -40.595243 |
| Brazil | Parque Nacional de Itatiaia, brejo da Lapa, Itamonte, MG | -22.289 | -44.868013 |
| Brazil | Parque Nacional de Itatiaia, brejo da Lapa, Itamonte, MG | -22.289 | -44.868013 |
| Brazil | Parque Nacional de Itatiaia, brejo da Lapa, Itamonte, MG | -22.289 | -44.868013 |
| Brazil | Parque Nacional de Itatiaia, brejo da Lapa, Itamonte, MG | -22.289 | -44.868013 |
| Brazil | Rio das Flores, Itamonte, MG | -22.289 | -44.868013 |
| Brazil | Parque Nacional de Itatiaia, brejo da Lapa, Itamonte, MG | -22.289 | -44.868013 |
| Brazil | Parque Nacional de Itatiaia, brejo da Lapa, Itamonte, MG | -22.289 | -44.868013 |
| Brazil | Parque Nacional de Itatiaia, near Brejo da Lapa, Itamonte, MG | -22.289 | -44.868013 |
| Brazil | Parque Nacional de Itatiaia, brejo da Lapa, Itamonte, MG | -22.289 | -44.868013 |
| Brazil | Parque Nacional de Itatiaia, near Brejo da Lapa, Itamonte, MG | -22.289 | -44.868013 |
| Brazil | Sítio Santana do Papagaio, Carangola, MG | -20.735 | -42.031181 |
| Brazil | Municipality of Camanducaiea, State of Minas Gerais, Atlantic rainforest | -22.7553 | -46.14472 |
| Brazil | Vila de Monte Verde, Municipality of Camanducaia, State of Minas Gerais | -22.8772 | -46.03383 |
| Brazil | Estação Ecologica Mina D'água, Apucarana, PR | -23.5413 | -51.382914 |
| Brazil | Morretes, in the Atlantic Forest | -25.3513 | -48.882148 |
| Brazil | Morretes, in the Atlantic Forest | -25.3513 | -48.882148 |
| Brazil | Reserva Particular do Patrimônio Natural Frei Caneca, Jaqueira, PE | -8.73898 | -35.793171 |
| Brazil | Reserva Particular do Patrimônio Natural Frei Caneca, Jaqueira, PE | -8.73898 | -35.793171 |
| Brazil | Itatiaia, RJ | -22.4957 | -44.560946 |
| Brazil | Teresópolis, RJ | -22.4123 | -42.956432 |
| Brazil | Teresópolis, RJ | -22.4123 | -42.956432 |
| Brazil | Teresópolis, RJ | -22.4123 | -42.956432 |
| Brazil | Reserva Biológica do Tinguá, Nova Iguaçu, RJ | -22.7598 | -43.451554 |
| Brazil | Paraty, RJ | -23.2167 | -44.717938 |
| Brazil | Parque Nacional de Itatiaia, Itatiaia, RJ | -22.4957 | -44.560946 |
| Brazil | Paraty, RJ | -23.2167 | -44.717938 |
| Brazil | Parque Nacional de Itatiaia, Itatiaia, RJ | -22.4957 | -44.560946 |
| Brazil | Parque Nacional da Tijuca, Rio de Janeiro, RJ | -22.9035 | -43.209587 |
| Brazil | Parque Nacional da Tijuca, Rio de Janeiro, RJ | -22.9035 | -43.209587 |
| Brazil | Parque Nacional da Tijuca, Rio de Janeiro, RJ | -22.9035 | -43.209587 |
| Brazil | Planalto do Itatiaia no Parque Nacional do Itatiaia , itatiaia, RJ | -22.322 | -44.590751 |
| Brazil | Santo Antonio do Pinhal, SP | -22.8254 | -45.66345 |
| Brazil | Estação Ecológica de Boracéia, Salesópolis, SP | -23.5325 | -45.846444 |
| Brazil | Apiaí, SP | -24.5137 | -48.843215 |
| Brazil | Reserva Biológica Serra do Japi, county of Jundiaí, state of São Paulo | -23.2494 | -46.949833 |
| Brazil | Reserva Betary, Iporanga, SP | -23.4392 | -47.424532 |
| Brazil | Parque Estadual Intervales, Ribeirão Grande, SP | -24.0979 | -48.371527 |
| Brazil | Reserva Betary, Iporanga, SP | -23.4392 | -47.424532 |
| Brazil | Estação ecologica de jataí, Luiz Antônio, SP | -21.5495 | -47.706674 |
| Brazil | Serra do Japi - Sítio do Sol, Cabreúva, SP | -23.3058 | -47.131187 |
| Brazil | Barão Geraldo, Campinas, SP | -22.9064 | -47.061574 |
| Chile | Santa Olivia, La Palma | -32.8759 | -71.17753 |
| Chile | Parinacota | -18.2039 | -69.26877 |
| Chile | Putre river | -18.1957 | -69.56901 |
| Chile | Caquena | -18.0627 | -69.20637 |
| Chile | Cosapilla | -17.7563 | -69.40875 |
| Chile | Charopalca | -18.0449 | -69.28153 |
| Chile | Allanes stream, close to Lluta river | -17.9958 | -69.62902 |
| Chile | Chungará lagoon | -18.2715 | -69.15636 |
| Chile | Putre | -18.196 | -69.57139 |
| Chile | Rio Limari site 1 | -30.667 | -71.516 |
| Chile | Quillota | -32.876 | -71.177 |
| Chile | Quebrada de Cordova | -33.433 | -71.65 |
| Chile | Las Chilcas | -32.868 | -70.843 |
| Chile | Rio Limari site 2 | -30.73 | -71.678 |
| Chile | Batuco | -33.197 | -70.841 |
| Chile | Laguna El Peral | -33.507 | -71.608 |
| Chile | Yali | -33.801 | -71.697 |
| Chile | Rapel | -34.175 | -71.474 |
| Chile | Yaquil | -34.563 | -71.481 |
| Chile | Rio Maipo | -33.633 | -71.533 |
| Colombia | near Ubaque, Departamento Cundinamarca | 4.436667 | -73.919444 |
| Colombia | Golondrinas, Belmira, Antioquia | 6.5956 | -75.6406 |
| Colombia | Quebradona, Belmira, Antioquia | 6.6379 | -75.6634 |
| Colombia | Quebradona, Belmira, Antioquia | 6.6379 | -75.6634 |
| Colombia | Santa Rita, Belmira, Antioquia | 6.5905 | -75.674 |
| Colombia | Golondrinas, Belmira, Antioquia | 6.5956 | -75.6406 |
| Colombia | Salema, Belmira, Antioquia | 6.5986 | -75.6569 |
| Colombia | Salema, Belmira, Antioquia | 6.5986 | -75.6569 |
| Ecuador | Rio Pucayacu, near Reserva Zanjarajuno | -1.37278 | -77.852222 |
| Ecuador | Rio Pucayacu, near Reserva Zanjarajuno | -1.37278 | -77.852222 |
| Ecuador | Rio Pucayacu, near Reserva Zanjarajuno | -1.37278 | -77.852222 |
| French Guiana | ADNG | 5.563611 | -53.944833 |
| French Guiana | Montage des singes | 5.075083 | -53.441469 |
| French Guiana | Matoury | 4.870833 | -52.352778 |
| French Guiana | Tresor | 4.607222 | -52.279444 |
| French Guiana | Favard | 4.505 | -52.045833 |
| French Guiana | Inselberg | 4.06825 | -52.689472 |
| French Guiana | Saut maripa | 3.810778 | -51.896111 |
| French Guiana | Parare | 4.045278 | -52.680278 |
| Peru | Quebrada Los Alisos, near Pataz, Provincia de Pataz, Departamento de La Libertad | -7.79222 | -77.59583 |
| Peru | Quebrada Los Alisos, near Pataz, Provincia de Pataz, Departamento de La Libertad | -7.79222 | -77.59583 |
| Peru | Site B, Condor Pass, above Laguna Sibinacocha, Cordillera Vilcanota | -13.8431 | -71.12244 |
| Peru | Site F, pond in deglaciated area in upper Laguna Sibinacocha watershed, Cordillera Vilcanota | -13.7518 | -71.08819 |
| Peru | Stream between Sina and Lusuni, Depto. Puno, Prov. Sandia | -14.5649 | -69.30075 |
| Peru | Lusuni, between Trapiche and Sina, Depto. Puno, Prov. Sandia | -14.5652 | -69.30106 |
| Peru | Between Santa Rosa and San Juan del Oro, Depto. Puno, prov. Sandia | -14.2136 | -69.13597 |
| Peru | San Miguel, Marcapata Valley, Depto. Cusco prov. Ouispicanchis | -13.4039 | -70.89936 |
| Peru | Ahuashiyacu | -6.4578 | -76.30843 |
| Peru | Nanay River | -3.68572 | -73.2835 |
| Peru | Area de Conservacion Privada Huiquilla, Amazonas | -6.38333 | -77.48333 |
| Peru | Area de Conservacion Privada Huiquilla, Amazonas | -6.38333 | -77.48333 |
| Peru | Area de Conservacion Privada Huiquilla, Amazonas | -6.38333 | -77.48333 |
| Peru | Seco River | -8.59656 | -76.08714 |
| Peru | Sauce | -6.72453 | -76.25318 |
| Peru | Bocatoma | -6.4585 | -76.34968 |
| Peru | Pond near tunnel | -6.43118 | -76.30882 |
| Peru | Lamas | -6.38493 | -76.51527 |
| Peru | Tahuayo | -4.17703 | -73.15365 |
| Peru | Iquitos km 23 | -3.9586 | -73.375 |
| Peru | Iquitos km 71 | -3.75067 | -73.33228 |
| Peru | Allpahuayo Mishana | -3.75067 | -73.28228 |
| Peru | public market, Cusco | -13.5211 | -71.9825 |
| Peru | San Pedro Market, Cusco | -13.5238 | -71.97128 |
| Peru | Manu Learning Center | -12.7893 | -71.39175 |
| Peru | Cueva de las Lechuzas | -9.32867 | -76.02715 |
| Peru | Tambopata Research Center | -13.1333 | -69.6 |
| Peru | Puente Maranura | -12.965 | -72.66568 |
| Peru | Satipo | -11.2767 | -74.64673 |
| Peru | Oxapampa | -10.5456 | -75.35835 |
| Peru | Huampal | -10.1883 | -75.57519 |
| Peru | Pozuzo | -10.0491 | -75.54059 |
| Peru | Aguaytia | -9.05734 | -75.66543 |
| Peru | Lake Yarinachocha | -8.32561 | -74.59022 |
| Peru | public market, Cusco | -13.5211 | -71.9825 |
| Peru | Huayatara, Site 20 | -13.4812 | -75.079655 |
| Peru | Huayatara, Site 19 | -13.4454 | -75.023188 |
| Peru | Huamanga-Vischongo (Abra Toccto), Site 8 | -13.3462 | -74.217847 |
| Peru | Huamanga-Vischongo (Abra Toccto), Site 7b | -13.3365 | -74.137862 |
| Peru | Pampas-Palmitos, Site 17b | -13.3319 | -74.860308 |
| Peru | Huamanga-Vischongo (Abra Toccto), Site 7 | -13.33 | -74.155954 |
| Peru | Huamanga-Vischongo (Abra Toccto), Site 6 | -13.3293 | -74.118166 |
| Peru | Huamanga-Vischongo (Abra Toccto), Site 9 | -13.3281 | -74.235679 |
| Peru | Pampas-Palmitos, Site 16 | -13.3041 | -74.781429 |
| Peru | Apacheta, Site 14 | -13.2915 | -74.48056 |
| Peru | Apacheta, Site 15b | -13.29 | -74.649743 |
| Peru | Apacheta, Site 15 | -13.2886 | -74.547997 |
| Peru | Apacheta, Site 13 | -13.288 | -74.449133 |
| Peru | Huamanga-Vischongo, Site 5 | -13.2826 | -74.077285 |
| Peru | Apacheta, Site 13b | -13.2767 | -74.427104 |
| Peru | Vinchos, Site 10 | -13.2747 | -74.343436 |
| Peru | Yucay, Site 4 | -13.1936 | -74.04894 |
| Peru | Torobamba, site 3 | -13.0538 | -73.950679 |
| Peru | Torobamba, site 2 | -13.0337 | -73.902979 |
| Uruguay | Cerro Verde Protected Area, Rocha Department | -33.9458 | -53.50861 |
| Venezuela | Maracay, Estado Aragua | 10.25639 | -67.57972 |
| Venezuela | Parc Nacional Henri Pittier (PNHP), Rancho Grande, Estado Aragua | 10.35278 | -67.68389 |
| Venezuela | PNHP, Estacion Biologica de Rancho Grande, Estado Aragua | 10.34944 | -67.68444 |
| Venezuela | Km 34 Maracay-Ocumare de la Costa rd, Estado Aragua | 10.38861 | -67.74639 |
| Venezuela | Km 29 Maracay-Ocumare de la Costa rd, Estado Aragua | 10.36972 | -67.73578 |

Table 1

Overview of the presence only (P.O) points used in the study herein to model and predict *Batrachochytrium dendrobatidis* habitat in South America
