# Supplementary figures and images for: Predicted Batrachochytrium dendrobatidis infection sites in Guyana, Suriname, and French Guiana using the species distribution model maxent
Source: PLoS One. 2022 Jul 14;17(7):e0270134. doi: 10.1371/journal.pone.0270134 (PMC9282542; doi:10.1371/journal.pone.0270134)

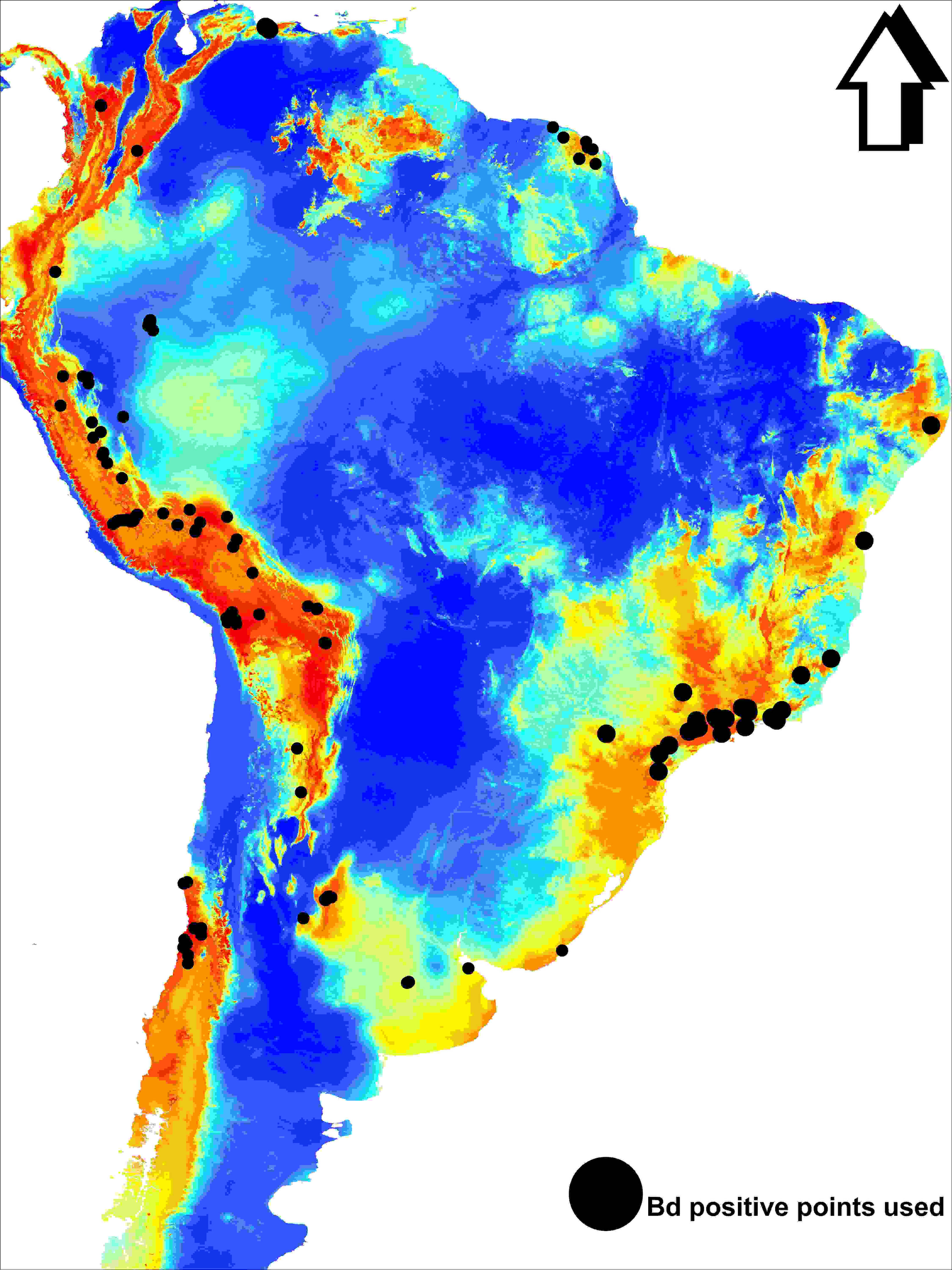

Supplement: S1 Fig — Overview of the presence only points used for this study as plotted by maxent Doi: 10.6084/m9.figshare.20024540. (TIF) [file pone.0270134.s002.tif]
